# Media technology is not the culprit: Factors influencing users’ quarrelsome behavior in political social platform use

**DOI:** 10.1371/journal.pone.0313099

**Published:** 2025-02-12

**Authors:** Zhong Fangqi, Lian Shuixing, Song Meijie, Yan Lingyan

**Affiliations:** 1 School of Communication, Fujian Normal University, Fuzhou, Fujian, China; 2 The Research Center for Marxism and Contemporary Media, Fuzhou, Fujian, China; Universidade de Santiago de Compostela, SPAIN

## Abstract

Social platforms are seen as hotbeds of political debate, where squabbles between users with different views and positions have become commonplace. From the perspective of social cognition, this study considered the individual variables (degree of political firmness and political efficacy) and situation variables (news credibility and life satisfaction) synthetically to construct a structural equation model. The purpose of this study was to explore the factors influencing users’ quarrelsome behavior in political use on social platforms. The results show that users’ political firmness, the credibility of news in the media environment, and real-life satisfaction all contribute to their quarrelsome behavior on social platforms.

## 1. Introduction

Social platforms have become important channels for the dissemination of grassroots and pluralistic views, attracting increasing numbers of people to participate in political discussions and public affairs online. In contrast to the traditional media model where people receive political information passively, the new social platforms give users more initiative, enabling them not only to receive information but also to actively produce, respond to, and share content. At the emotional level, research has shown that discussing public affairs on public–private social platforms can enhance people’s sense of security [1]. Today, the stream of political discussion on social platforms not only promotes political dialogue and participation but also embodies the spirit of public deliberation and thereby becomes an integral part of democratic institutions.

However, some academics have called for caution against social platforms giving the illusion of being in the public domain. In the case of computer-mediated communication, there are no real interpersonal norms or rules of conversation, which leads users to behave more recklessly when they are anonymous and irritate each other [2]. In recent years, text attacks on social platforms have become increasingly intense, with “flaming” frequently erupting [3]. Users are used to dissing, from the simple use of exclamation marks to the use of various emoticons to express mixed emotions, to the more serious language of confrontation, swearing, and intimidation [4, 5].

In particular, political or public affairs pages that reflect reality and involve multiple interests are the most impacted areas of conflict [6]. According to statistics, more than one-fifth of the comments on Facebook’s political news forum are offensive and derogatory [7]. Thus, although social media platforms provide cheap and convenient channels for the public to express its political opinions, in the media-oriented political environment, the emotional mobilization of political figures [8], online attacks and defense against online paid posters, and algorithmic conspiracies [9] may provoke irrational public sentiment and even spawn “digital populism”. Thus, social platforms may not be seen as the “public domain” of rational negotiation, but rather, they serve as a “pressure relief valve” for people to vent extreme emotions such as anger [10]. It is a reminder that while social platforms are an excellent way to get involved in politics, there is also a need to focus on their potential downsides.

Previous research in this area has mainly focused on certain aspects. Some studies have explored the environmental characteristics of social platforms related to conflict, like anonymity and lack of interpersonal norms. Others have considered individual factors such as political stance or efficacy, but not comprehensively considering both individual and situational variables simultaneously. There has been a lack of in-depth exploration of how these factors interact to influence users’ quarrelsome behavior during political participation on social platforms.

This study aims to fill this research gap by comprehensively considering both individual variables, namely political firmness and political efficacy, and situational variables, specifically news credibility and life satisfaction. By constructing a structural equation model, we aim to analyze the relationships among these factors. Our objective is to understand more precisely how these variables interact to affect users’ quarrelsome behavior on social platforms, providing a more comprehensive understanding and insights for effectively managing and guiding political discussions.

## 2. Literature review

### 2.1 Political use of social platforms: The battlefield of name-calling or the public domain?

The political use of social platforms refers to the use of the platform by users for political expression or political participation. Today, the concept of political participation is expanding; in addition to voting, marches, and other institutions within the framework of existing regulations, it is possible to use reasonable means to influence government decision-making—by institutionalized action, reading political articles, gaining political knowledge, tracking newspaper and television reports, and other indirect noninstitutionalized political participation [11]. It even includes the subjective components of attitudes, emotions, and perceptions that people reveal [12]. In short, the public has gradually changed from an “informed citizenship” to an “expressive citizenship” in the era of traditional media.

However, when people express their positions, they will inevitably have conflicts because of different ideas and interests. Numerous scholars have studied ideas of name-calling, slander, ridicule, and vulgar behavior in popular political participation [7, 13], which is defined as quarrelsome behavior in this study. Antagonism in political contexts has emerged in the era of traditional media. For example, political programs on television engage in deliberate debates in an uncivil tone to pique viewers’ interest [14]. This study found that people are more likely to see different political quarrels on the internet than they are in interpersonal communication, and they are more likely to engage in unbridled online attacks on opponents [15]. These attacks are often accompanied by negative emotions, the use of uncivilized content, being indiscriminate, and constantly belittling the blame of the resentment of the criticism, the most vivid expression of “electronic hatred” [16].

The reason is related to the socially inhibited behavior caused by the characteristics of social platforms. On the one hand, an individual’s anonymity in the online world protects their real identity, and users do not have to think too much about the real-world consequences of what they say when discussing politics. As a result, people tend to discuss matters online rather than in person when they need to vent their differences [17]. Online communication, on the other hand, is mostly about words or images. Lack of information leads to a lack of social cues, which, in turn, leads to a decrease in self-consciousness and self-control, and impulsivity and deviant behavior increase, indicating aggressive behavior. Dyer et al. [18] found that in computer-mediated communication, threatening words appeared four times more frequently than in face-to-face communication.

Moreover, political quarreling in online contexts is considered more harmful than in interpersonal contexts [19]. Research has confirmed that such wrangling leads users to develop negative perceptions of the quality, legitimacy, and credibility of news content [20], and exacerbates polarization of user attitudes toward public events and issues [21]. In the long run, not only does political trust suffers but also even threatens the established democratic system. Moreover, due to the lack of real-world judicial units, regulatory committees, and professional arbitrators to settle disputes on social media platforms [22], it is difficult to suppress speech conflicts effectively on the internet.

In summary, previous studies have focused on analyzing the causes of political quarreling from the perspective of the environmental characteristics of social platforms, and emphasized the negative impact of these political quarrels. However, there is little discussion from the perspective of the user as the main actor and the macro environment. What is the cause of the quarrels among users in the context of political participation on social platforms? This study adopts the perspective of social cognitive theory, which is widely used in the field of individual behavior research, wherein individual behavior is constructed by the environment, individual cognitive factors, and behavior itself. Therefore, from the perspective of users, to explore the dynamic interactions between media use, subject cognition (attitude, values), social environment (including daily life and media environment), and individual behavior (quarreling), we construct a mechanism for generating user behavior on social platforms. A breakdown of the factors is as follows.

### 2.2 Political stance: Social media platforms strengthen political attitudes

A political stance refers to the basic views and attitudes held by individuals in the field of social politics. It reflects the degree of an individual’s identification with and support of the social–political system, political phenomena, and political values. Internet technology has not created the global village of human solidarity as expected, instead, there is a growing tendency to split the online society into the “Cyberbalkans” [23] where a myriad of polarized and even bigoted opinion groups coexist. Sunstein [24], an academic who coined the term “group polarization,” goes on to point out that the internet world is twice as polarized as real life. Over the past few decades, as disagreements between universal political groups have widened, users of social media platforms have developed a strong emotional attachment to like-minded “Me groups.” Resentment and animosity toward dissenting groups have also gradually intensified [25]. Research has shown that audiences who rely mainly on social platforms for information tend to exhibit more assertive political attitudes than those who rely on traditional media such as newspapers and television [26]. This raises the following research hypotheses:

H1a: there is a positive correlation between political participation and political stance firmness on social platforms.
H1b: there is a positive correlation between political acceptance and political stance firmness in traditional media.

Existing positions largely determine how users respond to social platform speech. Cognitive defense mechanisms are activated automatically when users encounter speech that is inconsistent with their own positions. At this point, whether or not the speech is civilized and neutral, it will be judged as being poor quality and unconvincing, and users will retaliate [27]. In particular, on politically controversial issues, the assimilation of prejudice theory holds that reading information from different positions does not narrow the gap between those who hold different positions, as claimed by public deliberative views, but rather, widens the cognitive gap, leading to greater polarization [28]. Moreover, when users are more factional or take a stronger stand on an issue, they are more likely to develop irrational feelings of favoritism within a group and hostility outside a group [29]. Furthermore, users are more willing to express themselves more aggressively [30]. The following research hypothesis is proposed:

H2: There is a positive correlation between political stance firmness and online quarrelsome behavior.

### 2.3 Political efficacy: Social platforms promote political awakening

Social cognitive theory emphasizes that perceived self-efficacy is the most important and universal mechanism in individual behavior [31]. Angus Campbell, an American political scientist, proposed the concept of political efficacy as the feeling that one’s political actions can exert political influence on the political process. Using political efficacy as a measure of democracy, he argued that the more individuals believe that their political actions can influence the political process, the more motivated they are to exercise civic responsibility [32] In particular, when people’s values and dignity are embodied in political activities, their political efficacy will be strengthened [33]. In other words, individuals often derive their political efficacy from their direct or indirect experience in political activities.

In terms of media use, traditional research on political efficacy has confirmed that the more frequently people receive information on public affairs through media such as newspapers, magazines, radio, or television, the more they will have an immersive understanding of political events, and the greater their ability to participate in politics and the confidence to change the political status quo [34, 35]. At present, the new form of political participation represented by network politics is regarded as a kind of spiritual or experiential encouragement because of its advantages such as low cost, the quick response of the government, the thorough resolution of problems, and the intuitive effect on the sense of participation and political efficacy of the middle class [36], urban residents [37], college students, and adolescents [2] were further enhanced. The following research hypotheses are proposed:

H3a: there is a positive correlation between political participation and political efficacy on social platforms.
H3b: there is a positive correlation between political reception and political efficacy in traditional media.

However, the rise of social media has also brought new issues in the study of political efficacy. Although high political efficacy has undoubtedly increased people’s willingness to participate in the governance of social conflicts, it also brings a series of problems such as irrationality, disorder, and network violence in the pluralistic and free network environment [38, 39]. Especially on issues of high public concern and self-interest, such as healthcare, education, and income equity [36, 40], a sense of high efficiency has the potential to trigger anger and populist behavior [41]. This raises research questions:

Q 1: Is there a positive correlation between political efficacy and online quarrelsome behavior?

### 2.4 News credibility: The media environment influences user behavior

News credibility refers to the credibility of news organizations and news channels and how news content is perceived by the audience. This assessment is based on subjective perceptions of the length, frequency, type, and content of media use, rather than solely on the quality of the information itself [42]. Previous research has indicated that the public tends to regard media they prefer or use frequently as the most credible [43]. Traditional media are generally considered highly authoritative due to their good social reputation and brand influence accumulated over the years, and their content is often widely recognized by the audience [44]. In China, social media platforms such as Weibo and WeChat share the responsibility of disseminating political information with traditional state media and have a high degree of credibility in general, and together with other traditional media, they have become key channels for the public to obtain information [45]. The research hypotheses are thus presented:

H4a: There is a positive correlation between political participation on social platforms and news credibility.
H4b: There is a positive correlation between the political reception of traditional media and the credibility of news.

News quality is of great importance to society, not only for its own sake but also for its political implications. Not only is the media the main source for the public to obtain political information and form political cognition and attitudes but also the degree of public trust in the news shapes their political attitudes and behavior [46]. Past research has shown that the stronger the trust in news, the greater the confidence in social development [47], but other research has found that if the trust in online news is strong, individuals may perceive social problems to be more serious [48]. However, the current domestic research has not directly explored the impact of user-perceived news atmosphere on their network behavior. Therefore, the research question is

Q2: Is there a negative correlation between news credibility and online quarrelsome behavior?

### 2.5 Life Satisfaction: Social media as an outlet

Social cognitive theory emphasizes the important influence of environmental conditions on individual behavior differences. In the field of political behavior research, scholars generally believe that individuals should not be studied in isolation and that social factors such as the political and economic environment play a potential role in shaping individual political views and behavior [49]. There is no exact origin of the concept of life satisfaction, but it involves the individual’s overall evaluation and feeling about their quality of life. Nowadays, when people are faced with social pressure such as employment and housing issues, they tend to escape the norms of the real world through the internet and enter a fantasy world to seek spiritual liberation and comfort to make up for the inadequacies of real life [50].

In terms of political participation, the social frustration of people today often stems from the gap between the formation of their own economic and emotional needs and the satisfaction of their social needs [51]. People use the media to exert pressure on government decision-making through public opinion, or to satisfy their demands and interests, buffering the game between the people and the government [52] and thus enhancing people’s trust in the country and the government, promoting positive interactions between individuals and society [53]. The research hypotheses are thus proposed:

H5a: There is a positive correlation between political participation on social platforms and life satisfaction.
H5b: There is a positive correlation between political engagement and life satisfaction.

In addition, social discontent is an important factor driving people to participate in online political activities. Resentment against the social environment and political system not only has a powerful cathartic power but also may constantly erode the moral structure of individuals and distort their value order [54]. When this dissatisfaction reaches its limit or is stimulated by a particular event, it can erupts in the form of irrational aggression [55]. Research has shown that online quarrels are more satisfying than real-life ones, so the use of social platforms to vent real-life grievances is a current trend [22]. The research hypothesizes:

H6: There is a negative correlation between life satisfaction and online quarrelsome behavior.

## 3. Research methods

### 3.1 Data collection

In this study, secondary data were analyzed using the data collected by the research project of the Taiwan Communication Survey. The survey, titled “Communication and Civil Society—Citizen and Political Communication,” looked at Taiwan residents over the age of 18 with a local household registration, and those who lived at least four days a week at a sampled address were eligible for the survey. Interviews were conducted between July 11, 2022 and October 10, 2022 with a valid sample size of 2,015. The study had not access to information that could identify individual participants during or after data collection

### 3.2 Respondents

In the sample, 47.5% were male, 52.5% were female, 16.5% were 18–29 years old, 18.7% were 30–39 years old, 21.2% were 40–49 years old, 15.6% were 50–59 years old, 15.6% were 60–69 years old, 12.4% were 70 years old and over. Among them, 9.8% had completed primary school education, 9.4% had a junior high school education, 25.7% had a senior high school, technical secondary school, or equivalent education, 11.8% had a junior college education, 33.0% had a university education, and 10.3% had a postgraduate education.

### 3.3 Measurement

According to the literature, the preliminary structure of individual cognition and social environment impacting the quarrelsome behavior of social platforms has been confirmed. Therefore, based on the results of research motivation, research questions, and related literature analysis, this study summarizes the main variables, which are social media/traditional media political participation, political stance firmness, political efficacy, news credibility, life satisfaction, and online quarrelsome behavior. Among them, political participation by social media and the reception of political information by traditional media adopted the four-level Likert scale (1 = never, 2 = rarely, 3 = sometimes, 4 = often), with higher scores indicating more frequent use. Other variables were measured on a five-level Likert scale (1 = strongly disagree, 2 = disagree, 3 = General, 4 = agree, 5 = strongly agree), with higher scores representing stronger agreement attitudes. Specific variable constructions and measures as shown in Table 1.

**Table 1:** Variable constructs and measures.

Variable	Indicator	Measurement	Source
Social media political participation	SP1	How often do you search, browse, click, or watch news, information, or films related to “political or public affairs” on social media platforms?	Adapted from Boulianne [56] and Wolfsfeld et al. [57].
	SP2	How often do you repost, forward, or share news, messages, or films related to “political or public affairs” on social media platforms?	
	SP3	How often do you send “political or public affairs”-related messages (including making movies or commentary news) on social media platforms?	
Traditional media political reception	TP1	How often do you get political or public affairs information from newspapers?	Adapted from McLeod et al. [58] and Gil de Zúñiga et al. [59].
	TP2	How often do you get political or public affairs information from magazines?	
	TP3	How often do you get political or public affairs information from radio?	
	TP4	How often do you get political or public affairs information from TV?	
Political stance firmness	PA1	Is it possible to object to all the ideas put forward by someone who does not agree with you politically?	Adapted from Gerber et al. [60] and Wilson et al. [61].
	PA2	Is it possible to explain your position and listen to someone who does not share your political views to seek consensus?	
	PA3	Is it possible to work with someone who disagrees with you politically to find out why and how you might resolve your differences?	
Political efficacy	PE1	Does it matter who we vote for?	Adapted from Kushin & Yamamoto [62].
	PE2	Sometimes politics is too complicated, so do we ordinary people really not understand?	
	PE3	Do you agree that people like me cannot influence what the government does?	
News credibility	NR1	How credible is television news?	Adapted by Bucy [63].
	NR2	How credible is newspaper (print) news?	
	NR3	How credible is broadcast news?	
	NR4	How credible is magazine (print) news?	
	NR5	How credible is internet news?	
Life satisfaction	LS1	Overall, are you satisfied with your current family situation?	Adapted from Diener et al. [64] and Pavot et al. [65].
	LS2	Overall, are you satisfied with your current relationship status?	
	LS3	Overall, are you satisfied with your current financial situation?	
Online quarreling	AB1	When discussing politics on the internet, are you likely to leave a rude message?	Adapted from Buss & Perry [66].
	AB2	When you see people on the internet who disagree with you, is it possible for you to be blunt?	
	AB3	When you see different opinions on the internet, is it possible for you to leave a message to reason with those people?	
	AB4	If someone directly challenges your idea online, is it possible to fight back?	

## 4. Results

The study used PLS—SEM for several reasons. Firstly, it is suitable for exploratory research as it doesn’t require strict data distribution assumptions, allowing for flexible analysis and providing insights into variable relations [67]. Secondly, it effectively estimates complex relations and can handle both formative and reflective models, which aids in understanding the influence of predictors on the outcome and provides a comprehensive view of model relations. Finally, given a sample size of 2015, it is more suitable for smaller samples than CB—SEM and our sample size was within its reliable range [68]. SPSS29.0 and JASP 0.18.3 were used to process the data.

### 4.1 Measurement model

The reliability and validity of the scale were tested. First, the reliability test was performed, and, as shown in Table 2, Cronbach’s α coefficient and composite reliability (CR) were greater than. 60, indicating the reliability of the scale. Second, to test convergent validity, as shown in Table 2, the index factor loadings based on confirmatory factor analysis were above .50, below 95, p < .001. The average variance extracted (AVE) of social media platform political participation, political stance firmness, news credibility, life satisfaction, and online quarrelsome behavior were all higher than .50. At the same time, the AVE of traditional media political reception and political efficacy was greater than .40 and Cronbach’s α coefficient was greater than .60, therefore, the scale has convergent validity [69]. Third, to test the discriminant validity, the square root of the AVE for each concept in Table 3 was greater than the correlation coefficient between the concept and other concepts [69], and the values in the matrix in Table 4 were all less than 0.85 [70] according to the heterotrait–monotrait (HTMT) ratio, indicating that the model has good discriminant validity.

**Table 2:** Reliability measures.

Indicator	M	SD	Factor loadings	Cronbach’s α	CR	AVE
SP1	2.854	1.040	.699	.755	.766	.518
SP2	2.437	.989	.799			
SP3	2.448	.953	.649			
TP1	2.775	.899	.657	.711	.752	.407
TP2	2.329	.793	.513			
TP3	2.534	.941	.584			
TP4	3.052	.912	.513			
PA1	2.761	1.035	.571	.798	.821	.608
PA2	3.067	1.022	.943			
PA3	2.846	1.025	.843			
PE1	3.232	1.100	.690	.677	.685	.424
PE2	2.515	.997	.575			
PE3	2.757	1.124	.814			
NR1	3.205	1.029	.879	.913	.914	.628
NR2	3.236	1.022	.918			
NR3	3.128	1.017	.795			
NR4	3.101	1.021	.830			
NR5	2.838	0.982	.719			
LS1	4.081	.936	.702	.765	.766	.522
LS2	3.914	.897	.663			
LS3	3.460	1.072	.736			
AB1	2.110	1.043	.921	.885	.868	.647
AB2	2.156	1.012	.928			
AB3	2.399	1.150	.829			
AB4	2.310	1.115	.822			

**Table 3:** Discriminant validity.

	SP	TP	PA	PE	NR	LS	AB
SP	0.758						
TP	0.041	0.582					
PA	0.081	0.028	0.844				
PE	0.092	0.094	0.208	0.777			
NR	0.125	0.153	0.011	0.053	0.843		
LS	−0.034	0.051	−0.035	0.017	0.101	0.823	
AB	0.125	−0.022	0.187	0.089	−0.055	−0.105	0.858

**Table 4:** HTMT.

	SP	TP	PA	PE	NR	LS	AB
SP	1.000						
TP	0.126	1.000					
PA	0.173	0.100	1.000				
PE	0.133	0.096	0.339	1.000			
NR	0.033	0.116	0.038	0.043	1.000		
LS	0.069	0.037	0.023	0.022	0.120	1.000	
AB	0.362	0.041	0.368	0.150	0.103	0.244	1.000

### 4.2 Structural model and hypothesis testing

In terms of the overall fitness test, Χ2 = 936.885, df = 262, and p < .001, which was significantly affected by the large number of samples. As shown in Table 5, the model fits well. On the whole, the proposed model is supported by the research data.

**Table 5:** Model fit index.

Fitting index	Measurement models	Structural model
χ 2/df	3.253	3.576
CFI	0.953	0.945
TLI	0.945	0.937
SRMR	0.054	0.068
RMSEA	0.033	0.036

**Fig 1:**
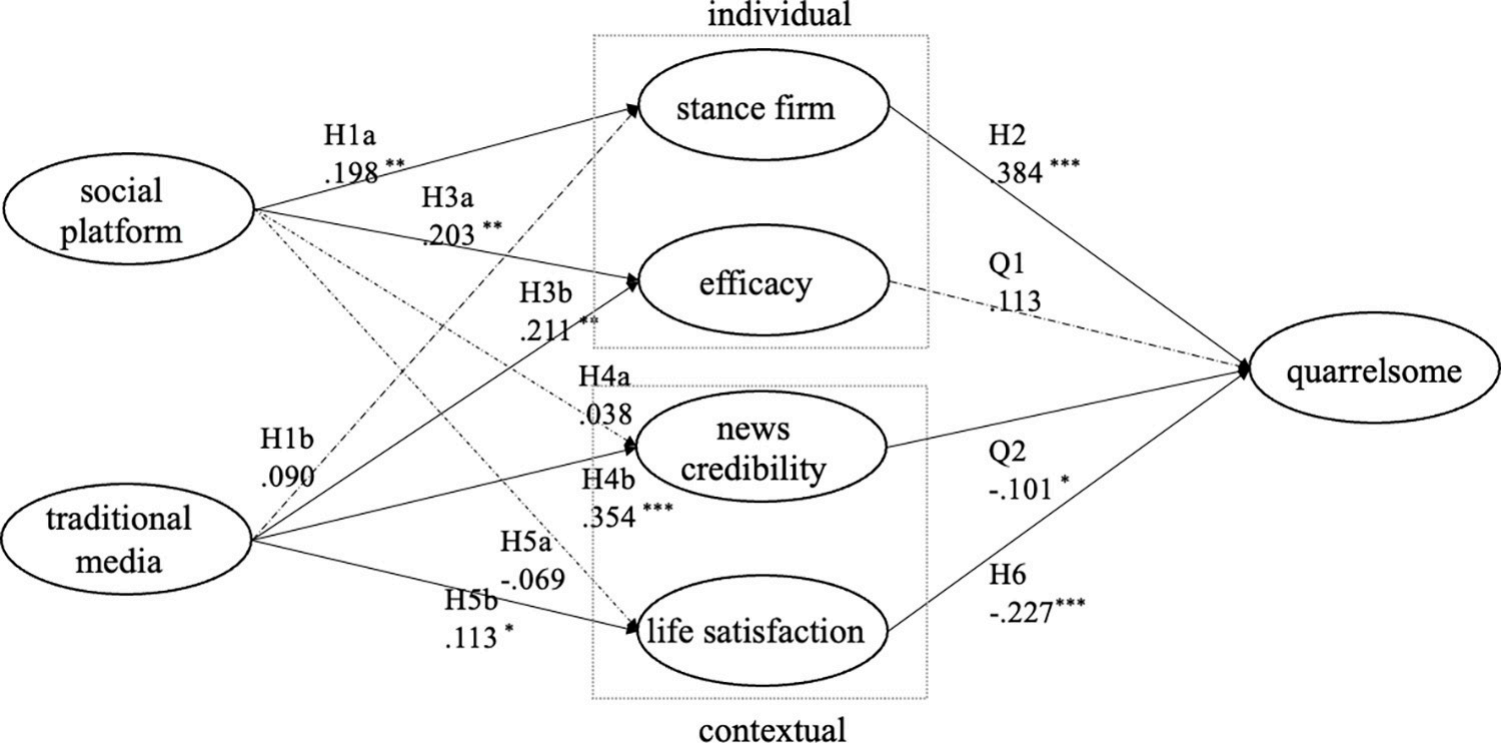
Path graph, a solid line with a significant path, * p < .05, * * p < .01, * * * p < .001.

For hypothesis verification, the path coefficients and significance of each hypothesis in the structural model were verified by the bootstrap resampling method (Resampling 5000 times). The results are as follows (Fig 1, Table 6): 1. Political participation on social media platforms (β = .198, p < .01) was moderately positively correlated with political firmness. However, using traditional media to receive political information (β = .090, P > .05) was not correlated with political firmness, that is, H1a was accepted and H1b was not. 2. There was a significant positive correlation between political stand (β = .384, p < .01) and online quarrelsome behavior, indicating that people with a more firm political stand were more likely to argue with netizens online, and H2 was, therefore, accepted. 3. Political participation of social media platforms (β = .203, p < .01) and the political reception of traditional media (β = .211, p < .01) were positively correlated with personal political efficacy, therefore, H3a and H3b were accepted. 4. There was no correlation between political efficacy (β = .113, P > .05) and online quarrelsome behavior, so Q1 was not accepted. 5. There was no correlation between political participation of social media platforms (β = .038, P > .05) and evaluation of news credibility, but there was a significant positive correlation between the political reception of traditional media (β = .354, p < .01) and news credibility, so H4a was not accepted, while H3b was accepted. 6. There was a slightly significant negative correlation between news credibility (β = −.101, p < .05) and online quarrelsome behavior, therefore, Q2 was accepted. The lower the credibility of news from different sources, the greater the likelihood of quarreling among netizens on the internet. 7. Social platform political participation (β = −.069, P > .05) was not correlated with life satisfaction, but political reception of traditional media (β = .113, p < .05) was slightly positively correlated with life satisfaction, so H5a was not accepted, while H5b was. 8. Life satisfaction (β = −.227, p < .01) was negatively correlated with online quarrelsome behavior. The lower the satisfaction with family, interpersonal relationships, and the economy, the easier it was to argue with netizens online, therefore, H6 was accepted.

**Table 6:** Hypothesis testing results.

Hypothesis	Relationship	Path Coefficient	Significance	Accepted/Rejected
H1a	SP—PA	0.198	p < 0.01	Accepted
H1b	TP—PA	0.090	P > 0.05	Rejected
H2	PA—AB	0.384	p < 0.01	Accepted
H3a	SP—PE	0.203	p < 0.01	Accepted
H3b	TP—PE	0.211	p < 0.01	Accepted
Q1	PE—AB	0.113	P > 0.05	Rejected
H4a	SP—NR	0.038	P > 0.05	Rejected
H4b	TP—NR	0.354	p < 0.01	Accepted
Q2	NR—AB	-0.101	p < 0.05	Accepted
H5a	SP—LS	-0.069	P > 0.05	Rejected
H5b	TP—LS	0.113	p < 0.05	Accepted
H6	LS—AB	-0.227	p < 0.01	Accepted

## 5. Conclusion and discussion

From the perspective of social cognition, this study comprehensively considered the factors of individual cognition and social environment and explored in depth the influential factors of online users’ quarrelsome behavior in the process of political participation on social platforms. The study found that political stance firmness, distrust of the media environment and dissatisfaction with real life are all important factors that aggravate users’ quarrelsome behavior on social platforms.

### 5.1 Hardening of positions: Social platforms breed determined political defenders

Originally, social platforms provided a forum for people to discuss political issues and engage with different political views, and in a sense acted as a “safety valve” for society, providing a new way for people to buffer class conflict and tension. However, the results of this study suggest that political engagement on social platforms only increases the politically entrenched position of users, which means that when users are exposed to different political views on social platforms, they may become more assertive and less willing to seek consensus or even compromise. Interestingly, in contrast, receiving political information through traditional media has no significant relationship with the consolidation of political stance firmness (H1A, H1B). This can be explained from the perspective of the internet’s “group polarization,” whereby the social platform’s environmental characteristics for the user’s choice of contacts, information, and political party color have provided unprecedented convenience, which greatly promotes the phenomenon of group polarization in the virtual world [71]. For example, the “group” function of a social platform provides an opportunity for like-minded users with similar interests and views to communicate, to take the user’s initiative of a “like attracts like, people separate” social network community to a new level; from a technical point of view, the “stand-as-market” strategy leads social platforms to provide users with information that fits their point of view, customizing the information environment for users through algorithms. To some extent, this deconstructs the pluralistic nature of social platforms, activating user identity boundaries and promoting both solidarity and exclusion within groups [72]; from a business perspective, social media platforms tend to prioritize short, emotive posts that provoke anger and continue to foment the issue, promoting unrestrained use of group polarization in pursuit of greater traffic and capital gains [73, 74].

The study also found that the more strongly a user takes a politically firm stance, the more likely they are to engage in an unfriendly quarrel with someone who does not agree with them when discussing politics on social platforms (H2). In the digital age, quarrels and conflicts are often no longer just disagreements based on opinions and rational arguments, but more about identity and emotional belonging [75]. Past evaluations of political online social communities in the United States [76] and Taiwan [77] have found that political discussion on the internet is clearly influenced by political preferences, where users tend to be segregated according to their ideology. People form the character of apathy and alienation in social relations in the environment of opposite groups, and it is difficult to listen to others’ views objectively and exchange opinions rationally [78]. In addition, the networked world also presents antagonism and conflict between various opinion groups, which greatly enhances the negative stereotyping and discrimination of individuals to the members outside the group. This may further create serious ideological divisions and antagonisms in society, rather than promote dialogue, with long-term effects leading to the growing popularization of democratic politics. What is more, group hatred on social platforms can be mirrored in the real world, triggering conflicting social events. Therefore, in future, social media platforms need to give more consideration to how to run discussion areas to reflect the value of public deliberation. For example, by strengthening the social communication ethics of users, we can guide them to release their emotions and opinions rationally, and eliminate “high participation willingness” and “low participation levels”.

### 5.2 Conscious effectiveness: Optimistic prediction of individuals’ benign political participation

The results of this study show that both traditional media and social media can effectively enhance the individual’s political efficacy (H3a and H3b). This is in line with previous research showing that media use increases feelings of efficacy [14, 79], which suggests that media not only provide people with opportunities to learn about politics and public affairs but also enhance their political awareness. It also provides the public with the means to influence government decision-making by expressing its views and demands, making the government more aware of its needs and interests, and enhancing its influence over the government.

Importantly, this study arrived at a reassuring conclusion: individuals with high political efficacy were not inclined to engage in quarrelsome behavior on social platforms (q 1). Presumably, individuals with high political efficacy have more ways to influence politics and government decision-making, so they are more likely to express their political interests in a rational, conventional way [80]. In contrast, “feelings of powerlessness,” as a key mediator of negative emotion formation, may be the root cause of resistance to active emotional expression, hate, envy, and impulsive behavior [54]. Thus, when people’s political efficacy improves, their interests are defended effectively, negative emotions are released, and irrational quarreling and catharsis may be suppressed. This finding suggests that we can safely raise people’s political awareness and provide them with a platform to participate in politics without excessive concern that large-scale political participation will lead to problems in maintaining stability.

In addition, some research shows that there is a circular effect between political participation and political efficacy. That is, not only do social media platforms for political participation increase political efficacy but the opposite is also true, in that increased political efficacy also significantly increases an individual’s willingness to participate politically on social platforms and their willingness to participate in offline political activities [81]. Therefore, we should further promote the two-way interaction and promotion between citizens’ political participation and political efficacy. For example, we should do this using social platform channels’ protection of the expression of interests, to enhance the responsiveness of the political system to the public and other measures, and at the institutional level to establish an information communication and buffer mechanism between the government and the public. As people improve their subjective assessment of their ability to perceive, participate in, and influence political activity, they may be more willing to express their opinions in a rational manner.

### 5.3 Media ecology: News distrust increases aggression

The study found, first, that people’s access to political and public issues through traditional media can improve the credibility of the news environment effectively, while social media platforms were not significantly associated with this evaluation (H4A and H4B). This may be due to several factors. Traditional media, such as television, radio, and newspapers, are regarded as authoritative channels for news transmission because of their strict procedures for news gathering, editing, and distribution, and as a result, people are more likely to trust their messages on political and public issues. In contrast, social platforms have more complex and diverse sources of information, including user-generated content, nonfactual opinions, and expressions of emotion, which can interfere with people’s overall assessment of the news environment. In addition, people may be influenced by cognitive biases in the way they process information, leading them to be more inclined to believe information that fits their point of view and to ignore or question information that contradicts it. This finding highlights the role of different media in information delivery and public perception, and reminds us of the impact of media choice and usage habits on public perception.

Second, an important finding of this study is that the lower the audience’s trust in the news environment, the more likely it is to have online quarrels (H6). This adds to the evidence that fake news harms democratic societies, which means that the dissemination of fake news not only weakens the dissemination effects of the news media but may also exacerbate social divisions and conflicts. In media ecology, the credibility of the news media is seen as an important cornerstone of a democratic system. However, with the diversification of information dissemination channels and the commercialization of the news industry, the credibility of the news media has faced unprecedented challenges. Currently, politically tinged fake news, especially disinformation and biased partisan information in the media, is on the rise [82]. Studies of the media during elections in various parts of the world have found that the media intentionally or unintentionally have the consequence of distorting public opinion and catalyzing political polarization during multiple elections [83, 84]. Therefore, maintaining the credibility of the media is closely related to the stability of democracy.

Moreover, from a cynical perspective, a high level of journalistic credibility can lead people to avoid online quarrels. Research shows that people’s trust in the media is positively correlated with cynicism, that is, the more they trust the media, the more likely they are to feel powerless to change society and the country [48]. This echoes the concepts of “powerlessness” and the “illusion of control” in social cognitive theory. Excessive trust in the media can lead to an over-identification with the status quo and a perception that everything is fixed and immutable, thus giving up on efforts to change the status quo. Such cynicism can exacerbate inequality and rigidity. It is, therefore, important to be alert to the risk of excessive trust in the media and to maintain the ability to think independently and critically to promote positive social change and development.

### 5.4 Environmental perspective: The key to a democratic society is not emerging technology

One of the important values of this research is that it successfully extends the network interpersonal behavior on the part of the virtual online community to real offline life, and establishes the close connection between the virtual and reality. The results suggest that access to political information through traditional media can improve life satisfaction, whereas political use of social media platforms cannot predict life satisfaction (H5A and H5B). The reason may be that traditional media, such as television, radio, and newspapers often undergo strict screening and examination when they disseminate political information, and the information they provide is more authoritative, comprehensive, and objective. Such authoritativeness and comprehensiveness enable the public to have a more accurate understanding of sociopolitical developments and policy trends when they obtain political information, thus contributing to a clearer and more rational political understanding, which, in turn, helps to improve people’s life satisfaction. However, there are problems with social media platforms, such as too much information generally and too little information to distinguish between what is real and what is not. The political information on social platforms is often mixed in with all kinds of entertainment and life information, which makes it difficult to attract the attention of the public. In addition, due to the anonymity and openness of social media platforms, some extreme political views are also easy to spread on them, thus misleading people’s political perceptions. This misleading and confusing political information environment may not have a positive impact on people’s life satisfaction.

In addition, this study also confirmed that real-life economic, family and interpersonal dissatisfaction will lead to users in the network environment being more likely to quarrel with others (H6). This may be because, against the background of increasing social structural contradictions, the solidification of the pattern of interest distribution and the functional defects in the process of government policy setting and operation have had an impact on public psychology, and this has led to the gradual accumulation and spread of grievances among the population [16] and more extreme and violent online expressions. In the past, the lower classes of society have used irrational acts such as jumping off buildings, self-immolation, and protest marches to conduct political resistance, expanding their demands into public discourse. Today, as social platforms serve as powerful channels for public voices, real-life grievances and struggles are reflected online in unison.

Continuing Shirky’s [85] “environmental perspective,” the key factor that drives the development of democracy and freedom is not the new technological tools themselves, but a strong civil society. A strong civil society can provide diverse sources of information and low-threshold opportunities for citizen interaction, which are important complementary tools for enriching the public sphere and strengthening civil society. Relevant data show that there is a close relationship between economic development and the political participation of citizens, and high-level political participation is always accompanied by high-level development. Social and economic development and prosperity provide a secure platform for people to participate in political activities [86].

Notably, the study also found a correlation between cyberattacks and real-life attacks, in which people who tend to attack others online may also exhibit aggressive behavior in real life. Therefore, to make social platforms play their political function better, and to prevent “electronic hatred” being mirrored in the real world to trigger conflict events, we need to focus not only on platform governance but also on the more fundamental level, that is, the elimination of social-transformation-induced resentment of structural factors and material incentives. The sustainable stability of the economy, the establishment and improvement of the legal system, the cultivation and development of civil society, and the improvement of the social–moral level can be fundamental to finding the key to social management.

In summary, this research not only comprehensively examines the interplay of individual and situational variables, a gap in prior research, but also uncovers relationships such as the impact of political stance firmness and life satisfaction on quarrelsome behavior that were previously less explored. These findings offer valuable insights for both platform management and public policy, guiding efforts to foster more rational political discussions and address social issues underlying user dissatisfaction.

## 6. Limitations and future directions

First of all, this study is limited by the database items used, and there is room for further improvement of the reliability and validity of some concepts, for example, the AVE of the potential variables “traditional media political reception” and “political efficacy” need improvement. Future research will further optimize its measurement to improve the AVE, such as refining measurement items and using more targeted data collection methods. Second, this study did not subdivide the credibility degree of traditional media/network new media news, but according to previous research, there are different causal relationships between news credibility evaluation and political participation by different media carriers [48].

Finally, future research may consider more factors to be included in the analysis of the influencing factors of quarrelsome behavior. For example, individual variables such as demographics and personality; content variables such as text analysis of messages, polarizing content and controversial material, especially religious issues and political party differences are considered likely to trigger intense emotions [87, 88]; atmosphere variables such as the spiral of silence hypothesis; and contemporary media variables such as Omnivore.
